# Perforating ocular fishhook trauma: a case report

**DOI:** 10.1111/cxo.12587

**Published:** 2017-09-06

**Authors:** Xiaohu Ding, Zhenzhen Liu, Ying Lin, Yangfan Yang

**Affiliations:** ^1^ State Key Laboratory of Ophthalmology, Zhongshan Ophthalmic Center Sun Yat‐sen University Guangzhou China

Fishing is a popular recreational activity world‐wide but it is occasionally the cause of very severe ocular trauma. There have been several reports demonstrating that trauma caused by fishing may involve the eye lid, cornea, sclera, anterior chamber and even the posterior vitreous.^1^ Here we describe a case of perforating fishhook ocular trauma that involved both the cornea and anterior sclera.

## CASE REPORT

A 34‐year‐old man presented to our emergency clinic on 28 June 2016 at midnight, with the chief complaint of trauma to his left eye by a fishhook occurring at approximately 20:00 hours. After three transfers without receiving any treatment, he arrived at our clinic at 2:00 hours.

**Figure 1:**
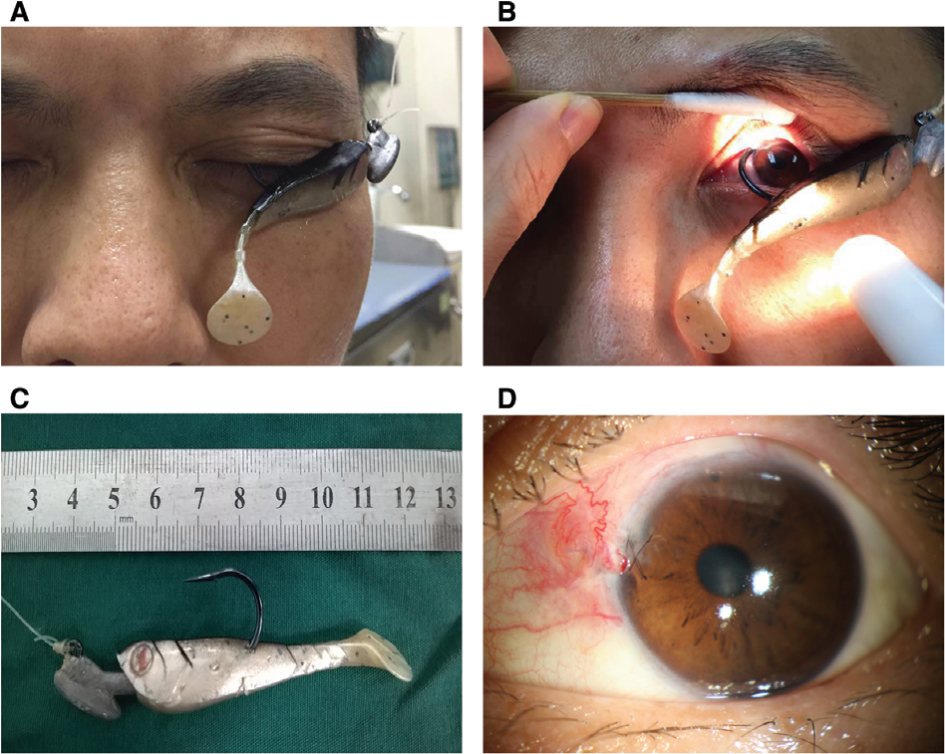
The appearance of the eye before and after surgery

Figure 1A shows the artificial bait that was present on his left eyelid. After retracting the upper eyelid, the eyeball was found penetrated at the superior‐nasal limbus (Figure 1B). The bend and shank were outside the eyeball, while the point was not visible (Figure 1B). Visual acuity in the left eye was 6/6. Slitlamp examination showed that the barb of the fishhook was trapped within the corneal stroma. Fibrinous exudates were found in the inferior anterior chamber. After effective communication with the patient, an emergency operation was performed under retrobulbar anaesthesia.

During the operation, we used a speculum to pull the upper lid. We found that the wound was located at 10 o'clock, just at the limbus. The fishhook point was not visible. Because the barb of the fishhook was hindered by the corneal stroma, we used a back‐out technique to remove the fishhook by enlarging the wound with a 20‐G needle. After smoothly pulling out the fishhook (Figure 1C), the anterior chamber disappeared and the iris prolapsed. We sutured the wound with interrupted 10‐0 nylon. We used one per cent pilocarpine to reposition the iris, and the anterior chamber was irrigated with balanced salt solution (BSS). We tried to reform the anterior chamber but this attempt failed.

We opened the superior conjunctiva to explore the superior sclera and found another wound at 12 o'clock on the anterior sclera. We sutured this scleral wound with 7‐0 Vicryl, and the anterior chamber then reformed. After the operation, systemic antibiotics were used for three days, and topical antibiotics and steroids were used for two weeks. Visual acuity in the left eye was 6/7.5 on post‐operative day one. One month later, visual acuity had reached 6/6 and a good anatomical result was achieved (Figure 1D). No complications were observed at three‐ and six‐month follow‐up appointments, and visual acuity remained stable.

## DISCUSSION

A prompt, appropriate surgical intervention is crucial for a good outcome.^2^, ^3^, ^4^ In this case, we used back‐out technique to remove the fishhook after enlarging the entry wound by a 20‐G needle. The technique should be carefully chosen by taking into account the type of fishhook, the depth of the fishhook and the relationship between the fishhook and related ocular tissue.

Fishing is a potential cause of eye trauma and all people who fish must be careful. It has been reported that trauma caused by fishing‐related injuries accounts for 20 per cent of all sports‐related ocular trauma in the United States.^1^ In our case, a latent scleral wound coexisted with an apparent corneal wound. The possibility of this type of injury should be taken into account in cases of fishhook trauma.

## Supporting information


**Video S1.** Video showing the entire operation.
