# Supplementary material for: Perforating ocular fishhook trauma: a case report
Source: Clin Exp Optom. 2017 Sep 6;101(2):297–8. doi: 10.1111/cxo.12587 (PMC5873264; doi:10.1111/cxo.12587)
Supplement: Supplementary file 1 — Video S1. Video showing the entire operation. [file CXO-101-297-s001.zip › vedioS1.docx]

**SUPPLEMENTARY INFORMATION**

**Video minutes**

[00:00–00:10] The whole fishhook was shown under microscopy.

[00:11–00:17] A lid retract was used to help open the upper lid.

[00:18–00:28] The cornea was incised superficially to enlarge the pathway for the removal of the fishhook.

[00:29–00:39] The barb was hooked in the corneal stroma and thus prevented removal of the fishhook.

[00:40–00:45] The corneal stroma, which had been stuck by the barb, was incised.

[00:46–00:52] The fishhook was pulled out smoothly using a pair of straight hemostatic forceps.

[00:53–01:02] The conjunctiva was opened and no scleral wounds were detected near the corneal wound.

[01:03–01:27] We began to suture the cornea with an interrupted 10-0 nylon at the limbus.

[01:28–01:48] We injected 1% pilocarpine into the anterior chamber to induce miosis. The iris was repositioned successfully.

[01:49–01:54] The knot at the limbus was tightened.

[01:55–02:01] We used balanced salt solution to replace the pilocarpine in the anterior chamber.

[02:02–03:14] The corneal wound was sutured with two additional stitches.

[03:15–03:39] The conjunctiva was opened superiorly. A penetrating scleral wound was detected at 11 o’clock, 3 mm posterior to the limbus.

[03:40–04:05] The scleral wound was sutured using 7-0 Vicryl.

[04:06–04:54] The knots were tightened and buried.
